# Road Condition Monitoring Using Smart Sensing and Artificial Intelligence: A Review

**DOI:** 10.3390/s22083044

**Published:** 2022-04-15

**Authors:** Eshta Ranyal, Ayan Sadhu, Kamal Jain

**Affiliations:** 1 Department of Civil and Environmental Engineering, Western University, London, ON N6A 3K7, Canada; eranyal@uwo.ca; 2 Department of Civil Engineering, IIT Roorkee, Roorkee 247667, India; kjainfce@iitr.ac.in

**Keywords:** road condition monitoring, pavement distress evaluation, pavement monitoring, smart sensors, AI, deep learning, machine learning

## Abstract

Road condition monitoring (RCM) has been a demanding strategic research area in maintaining a large network of transport infrastructures. With advancements in computer vision and data mining techniques along with high computing resources, several innovative pavement distress evaluation systems have been developed in recent years. The majority of these technologies employ next-generation distributed sensors and vision-based artificial intelligence (AI) methodologies to evaluate, classify and localize pavement distresses using the measured data. This paper presents an exhaustive and systematic literature review of these technologies in RCM that have been published from 2017–2022 by utilizing next-generation sensors, including contact and noncontact measurements. The various methodologies and innovative contributions of the existing literature reviewed in this paper, together with their limitations, promise a futuristic insight for researchers and transport infrastructure owners. The decisive role played by smart sensors and data acquisition platforms, such as smartphones, drones, vehicles integrated with non-intrusive sensors, such as RGB, and thermal cameras, lasers and GPR sensors in the performance of the system are also highlighted. In addition to sensing, a discussion on the prevalent challenges in the development of AI technologies as well as potential areas for further exploration paves the way for an all-inclusive and well-directed futuristic research on RCM.

## 1. Introduction

Roadway infrastructure is susceptible to structural degradation on account of material deterioration primarily caused by heavy traffic, harsh weather conditions, aging, poor construction quality, and lack of appropriate maintenance. The success of a road transport system is inherently dependent on the riding quality and comfort level of the commuters, for which timely detection of faults and ensuing maintenance is of utmost importance. The lack of rapid and automated road monitoring methods is a major contributing factor to pavement damages and is a common issue in many countries, necessitating huge efforts in this direction. Most developed countries witness a significant portion of aged pavements, while some suffer from deteriorated road networks due to extreme climatic events. On the other hand, developing countries suffer from pavement distress due to a radical increase in the number of vehicles and intensive usage. The manual observation and detection methods, though in practice, are cumbersome and time-consuming, involving high costs. During recent decades, researchers and engineers have prioritized safety and reduction in inspection costs in the scope of developing an intelligent and smart roadway infrastructure system to expedite maintenance and corrective action. Therefore, the significance of cost-effective road condition monitoring (RCM) systems to warrant long-standing structural integrity and safety levels has been emphasized globally. Emerging RCM methods have the potential to rationalize periodic inspections and minimize the costs associated with failing pavement structures. This paper aims to provide a systematic and comprehensive review of the latest advancement in AI-assisted RCM technology.

Pavement failure can be attributed to diverse factors, such as vehicle loading, environmental conditions, construction quality and maintenance. Some of the most commonly occurring pavement distresses are listed in Table 1. Vehicle loading and environmental conditions majorly contribute to surface disintegration, resulting in potholes, which become severe as disintegration moves down the layers with time. Cracking, which is another common form of pavement failure, occurs in different shapes and sizes, which can again be attributed to vehicle load, causing damage in areas with an inadequate packing of base layers and/or poor drainage, or poorly constructed paving lane joints or volume changes in the subgrade. There are other forms of distresses that come under pavement distortions, such as shoving, rutting, corrugation, depressions, and bumps and sags, which are observed parallel to the direction of traffic and occur due to a weakened pavement layer, poor bonding between layers, or excessive moisture in the subgrade. Surface deterioration is also observed in the form of raveling, weathering and bleeding owing to aging, vehicle loading, poor mix, poor compaction, dust, and moisture. Often, sealants and patches, which are preventative maintenance actions, appear as distresses over the course of time.

A typical RCM method relies on the data acquisition system that involves 1D time-series data, 2D visual data or 3D depth data. Low-cost sensors, such as accelerometers, gyroscopes, magnetometers, and GPS, are often employed to measure motion, rotation, velocity, orientation, and location, which are one-dimensional in nature. The commonly used sensors for acquiring digital images are the high-quality, high-resolution RGB sensors, which facilitate in acquiring multiscale low-level as well as high-level feature extractions. However, these images are two-dimensional in nature and cannot be used for the characteristic evaluation of the features. The depth consideration of such images allows multi-fold parametric attributes facilitating the examination of intrinsic characteristics and can be acquired by using thermal imaging sensors, LiDAR, laser sensors and GPR, to name a few. Table 2 provides a comparative evaluation of the next-generation sensors that have evolved the entire data acquisition process for RCM. The convenient deployment of these sensors on the ground and aerial platforms (i.e., drones) provides them with an edge in terms of scalability and employability. Table 3 illustrates the merits and limitations of different platforms on which sensors are commonly deployed.

Advances in next-generation sensors [1] and data-driven technologies [2,3,4] have transformed data collection and interpretation, contributing to extensive research on RCM. Recent years have seen the widespread use of vision-based approaches to provide cost-effective solutions in identifying and localizing pavement anomalies. Diverse methods have been presented, ranging from traditional image processing techniques to vision-based AI approaches. The archetypal vision-based RCM is commonly carried out either by employing image processing techniques (IPTs), such as thresholding, or by using machine learning (ML) algorithms for local feature extraction. In the process of implementing IPTs, encouraged by grayscale and texture-based methods, Ref. [5] performed asphalt pavement pothole detection and segmentation using a wavelet energy field to highlight the pothole region. The proposed method exhibited satisfactory performance with an overall accuracy of nearly 87%, and successfully differentiated the potholes from the cracks, patches, greasy dirt, shadows, and maintenance hole covers, and precisely segmented the pothole. However, the overall methodology was time consuming and required tedious processing.

Ref. [6], in his attempt to classify a distressed area, developed a unique two-stage RCM system. In the first stage, the artificial bee colony (ABC) algorithm was used to obtain a threshold value to segment the image into distressed and non-distressed sections, followed by feature extraction. The second stage involved training on a single-layer artificial neural network (ANN). Although the proposed method successfully classified an area of distress, there was a redundant and computationally intensive step to segment the images using the ABC algorithm, as the neural network alone can be efficiently trained to segment the images and extract features for classification. Ref. [7] proposed an unsupervised and automated pavement crack detection based on the analysis of photometric information from two-layer (intensity, depth) images. The method is based on histograms and Otsu’s thresholding. The results showed that the proposed method could be used for fast and approximate crack estimations, especially in cases of a low signal-to-noise ratio. Despite the limitations of the conventional IPTs in terms of handling the background complexities and illumination complications, continuous research has been carried out using IPTs to enhance and amplify the results. Recently, Ref. [8] proposed a three-phased crack detection system based on IPTs. The approach involved contrast enhancement followed by the application of a discrete wavelet algorithm for effective detection and, finally, Jerman enhancement to enhance crack detection. Comparisons with existing methods showed the effectiveness of the proposed technique to validate the recognition of surface cracks.

To improve the accuracy of existing RCM methods, ML approaches, such as KNN, SVM, and k-means clustering, were integrated with IPTs. Ref. [9] used ML-based pattern recognition methods with computer vision methods on street view images for the assessment of pavements. A series of IPTs followed by SVM [10] classification successfully identified the severity and location of pavement defects in the image. A comparison with the U-Net DL model on the same dataset suggested inferior performance due to scarce data. Ref. [11] presented a model based on the library of SVM (LIBSVM) to distinguish the potholes from the cracks in concrete pavements. The proposed model is effective in segmenting potholes with a high F1 score of 98.7%. A comparison with the Otsu, edge detection, k-means, and watershed methods re-establishes the processing efficiency of the method on cement concrete pavement potholes. 

Ref. [12] adopted a *k*-means clustering algorithm followed by Otsu’s thresholding to segment cracks in asphalt pavements. The proposed algorithm provided a satisfactory result in localizing and detecting pavement cracks. Ref. [13] developed a texture-independent, tile-based image-processing algorithm to detect pavement cracks and classify them into longitudinal and transverse cracks on 2D and 3D pavement images. The lengths of the cracks were measured using curve fitting and an orientation axis. The method suggested promising results when tested on 130 images of Portland cement concrete and asphalt concrete surfaces, and took less than 20s per image to generate results. Ref. [14] adopted an integrated approach, establishing IPTs in association with ML algorithms for automated road crack detection and classification. Heuristic segmentation and denoising algorithms were applied, followed by image-processing practices for enhancement and feature extraction, and the application of a multi-stage hybrid model based on ensemble learning for classification. A comparative study with existing ML approaches underscored the competitive performance of the proposed work.

In the past few years, there have been several review papers published in the field of RCM. For example, Ref. [15] performed an extensive review on the RCM methods focusing largely on the types of pavement distresses and the detection equipment used. A quantitative analysis of the work conducted in the past on individual pavement distresses, such as cracks, potholes, surface deformations and defects, and the macrostate of the road, such as road roughness, skid resistance, substructure quality and road surroundings, were performed. Different equipment, such as a camera, laser, accelerometer, acoustic, pressure sensor, radar, ultrasonic, deflectometer, friction tester for distress detection and data collection, are emphasized and compared in the study. The paper presents a detailed discussion on the various distresses and detection techniques; however, it does not focus on the algorithms used for RCM. Another substantive review by [16] highlighted the advancement of deep learning (DL) methodologies in accurate pavement crack detection. The DL techniques were catalogued under three headings: classification, object detection and segmentation. The performance metrics employed to evaluate these methods on benchmark datasets in addition to traditional and DL-based crack-detection methods on 3D data were also reviewed. In another review paper, Ref. [17] assessed the various computer-vision techniques employed in the detection of cracks and potholes in pavements. The techniques were categorized under DL, non-DL, depth camera and tire pressure/vibration methods. Varying inputs, such as RGB images, laser and thermal images for vision-based techniques, were discussed and evaluated. It was deduced that major detection techniques were oriented around computer vision approaches, though they failed to produce good results. RGB images owe their limitations to lighting conditions, texture variation and background complexity; however, laser and thermal imaging overcome these limitations, but suffer due to environmental and cost factors as well. Limitations in terms of the system size, computational requirements and real-time applicability were also highlighted in the study. Unlike DL methods, [18] in their review, summarized the research carried out on digital images in RCM based on IPTs and acquisition devices. The image acquisition devices, along with their characteristics, were compared, and various image technologies applied in pavement detection were expounded. Concurrently, the IPT for specific problems, such as on-site pavement cracks, pavement texture, rutting and potholes, was elaborated. The authors concluded that the salient factors in determining the detection equipment should be based on cost, efficiency, clarity of image details and type of pavement, while ML methods outperform the traditional IPT in RCM. 

Over the last few years, vision-based approaches have augmented RCM solutions and remarkably advanced the development of pavement monitoring and analysis. It is imperative to note that there are other existing reviews [15,16,17,18] addressing pavement distress studies, as discussed above. These review articles largely emphasize the types of pavement distresses, the detection equipment used, the sensor technologies available, and various computer vision techniques employed in the detection of cracks and potholes in pavements; however, they do not focus on the data-driven algorithms used for distress detection. The review on pavement-defect detection methods based on DL by [16] provided a substantive overview of the subject, but has a restricted scope. The proposed review in this paper aims to provide an all-inclusive review by focusing on the next-generation sensing technologies and associated AI-based RCM methods, by elucidating the methodologies and challenges in current developments, as well as recognizing the prevailing research voids for further research studies. Therefore, the criteria for consideration of research articles in this paper are the evaluation of the existing sensor-based and AI approaches deployed on different platforms, namely UAVs, ground vehicles and smartphones for RCM. AI is a data-driven amalgamation of various ML and DL algorithms inherently dependent on sensors and data acquisition to provide solutions to real-world problems. These algorithms are a subset of the entire AI domain and have been shifted from handcrafted feature extraction-based ML methods to automated DL methods. To help researchers and engineers better understand the application of AI methodologies in pavement monitoring and analysis, the current review summarizes the recent work that has been established from the year 2017 to 2022. The AI approaches are classified under sensor-based methodologies, along with the application of machine learning and deep learning algorithms for RCM. An exhaustive review of the DL methodologies is presented that builds on classification, segmentation and detection, employing next-generation sensors integrated with different data acquisition platforms.

This paper is organized as follows. Section 2 presents a brief discussion of the reviewed articles and their limitations as a comprehensive and systematic review. It elaborates on AI technology in general, categorized under next-generation sensors deployed on data acquisition systems and compares the related work in terms of the methodology adopted and results achieved. Section 3 presents the potential future research directions. Conclusions and challenges in using DL approaches in RCM are finally presented in Section 4.

## 2. Smart Sensing of Pavement Distress Data

**Figure 1:**
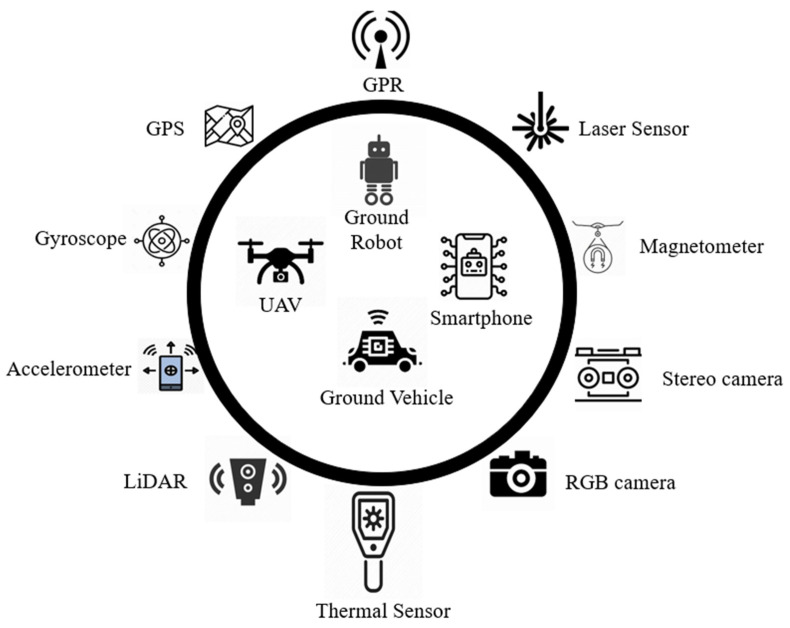
A schematic representation of next-generation sensors and their platforms.

Smart sensing techniques provide high-resolution information on pavements. Faster installation and flexibility in deploying noncontact sensors on various mobile data acquisition platforms facilitate convenient ways to inspect pavements, overcoming space and environmental constraints. Every platform has its advantages and limitations and complements each other’s usability. A schematic representation of the sensors and data acquisition platforms is shown in Figure 1.

Smart sensors primarily result in 1D (i.e., vibration time series) and 2D/3D (i.e., vision) RCM data, which were analyzed using various signal processing, machine learning, and AI techniques in the literature, as presented below.

### 2.1. Vibration Data-Based RCM

Smartphones are installed with diverse sensors, such as gyroscopes, accelerometers, and GPS receivers, and thus act as powerful measuring tools. Hence, capitalizing on the in-built vibration sensors to assist RCM has taken center stage in numerous research works. Vibration-based methods for detecting road anomalies rely on 1D data acquired from these sensors and find high applicability in the real-time detection of pavement distress due to low cost, fewer memory requirements and immunity to light conditions.

Ref. [19] used smartphone accelerometers to measure acceleration data. Basic signal processing and pattern recognition methods were used to correlate the measured data with the international roughness index (IRI), validating that variance in acceleration measurements can be used to determine IRI using smartphone sensors. Ref. [20] proposed an asphalt pavement quality monitoring system using data collected from accelerometer sensors of smartphones by treating the data as a multi-dimensional time series classification problem. The proposed approach attained a good classification accuracy. Ref. [21] compared a standard inertial profiler with a smartphone-based application, Roadroid, to establish the usability of smartphone sensors in estimating the ride quality of the concrete pavement. It was observed that Roadroid significantly underestimated the IRI values, though a linear correlation existed between the IRI measurements obtained from both methods. In another attempt, Ref. [22] proposed an AI-assisted smartphone-based data-driven technique to detect vibration-induced road anomalies using vibration sensors and the detection of pavement patch defects using smartphone images. The results showed that vibration-based methods were quite successful. However, they failed to efficiently cover the entire roadway and detect non-vibration-induced pavement anomalies, which were then complemented by a vision-based method.

Ref. [23] presented an AI-based low-cost RCM system by detecting pavement potholes using smartphone sensors and onboard diagnostic devices. The method is based on the observed patterns of the vehicle’s interaction with the road surface and confirms the usage of smartphone sensors in low-cost pothole detection. Ref. [24] provided a cost-effective pavement evaluation system for low-volume roads using smartphone-based roughness data. Compared to conventional roughness measurement methods, it was found that a good correlation existed between the results, when compared to the results from the Class III-type roughness measurement equipment, justifying sufficient accuracy to determine the presence of pavement distresses. Ref. [25] developed an RCM technique by using 3D pavement data to train ML models on low-cost vehicle-mounted smartphone sensor data. The model provided distress values as outputs, which can be used for estimating IRI. The applicability of the technique could be justified by a high peak in the correlation between IRI estimations produced from numerous runs along the same route. In another study, Ref. [26] proposed a method to determine the IRI of a pavement surface using conventional vehicles and smartphones by using the grey box model algorithm and the quarter-car vehicle model. The results indicate that the IRI of the pavement can be determined with reasonable accuracy using smartphones.

In a novel attempt, Ref. [27] proposed a vibration-based RCM system by considering factors, such as vehicle suspension and a direct reconstruction of the pavement profile, to compute IRI rather than using correlation-based procedures. Field testing of the method proves its potential for RCM with reasonable accuracy and efficiency. Ref. [28] used low-cost smartphone accelerometer data by developing a prototype smartphone application to measure the IRI, by incorporating vehicle speed and vehicle type. The results showed a strong correlation with the measured IRI data collected using profiler vans. Ref. [29] proposed a low-cost method employing smartphone sensors for pavement roughness measurement by collecting the vertical vibration values when a vehicle is driven over a pavement. The smartphone-based acceleration values are converted to the IRI by using the quarter-car simulation model. The high correlation values promise a model that can be used in smartphone-based RCM. Ref. [30] proposed a road condition tool (RCT) to assess road pavement defects based on a telemetric data crowdsourced by smartphone users by analyzing the vehicle motion dynamics. The results obtained, in comparison with reference data from highly specialized equipment, confirm the proposed RCT solution [31] established an algorithm to calculate the IRI from the acceleration values obtained using smartphone sensors. The algorithm identified the physical parameters of a quarter actual vehicle model and established a relation between the acceleration, IRI and the profile elevation. It was observed that a consideration of the dynamic characteristics of vehicles improved the proposed method’s measurement accuracy.

Thus, vibration-based methods find immense applicability in RCM. They provide a comparative measure of the degraded pavement condition that is memory friendly and suitable for the real-time detection of road anomalies, but are vulnerable to errors due to noise and signal sensitivity from other vehicle sensors. Moreover, the vibration effects caused by any road obstacle can be perceived as a road anomaly by these sensors. Additionally, it is important to note that vibration-based sensors only take into account data along the wheel path.

### 2.2. Vision Data-Based RCM Using Machine Learning

The exploitation of ML methods allows for the effective identification, classification and analysis of pavement structure conditions. Generally, ML approaches in pavement engineering comprise support vector machines (SVMs) and artificial neural networks (ANNs). The following section briefly summarizes the conventional ML approaches used in recent years in RCM using different data acquisition platforms.

#### 2.2.1. UAV-Based Data Acquisition

Multispectral image-based ML algorithms were employed by [32,33] on drone-acquired images to distinguish undamaged pavement from damaged pavement. SVM, ANN, and Random Forest (RF) algorithms were evaluated and compared both on RGB and multispectral images. It was deduced that, amongst the three algorithms, RF performs best with a high accuracy on multispectral images. In addition to the feature set, it was deduced that the spatial resolution of the pavement imagery is also a conclusive factor in the performance of the classifier. Ref. [34] developed an RCM system based on drone images using image-based methods to identify three types of cracks in pavements. Decent results were achieved by the proposed combination method developed by combining Canny edge detection and Otsu thresholding. Ref. [35] proposed the usage of low-flying UAV acquired multispectral imagery in combination with CNN extracted spatial features to assess the aging and damage conditions of pavements with the help of an SVM classifier. A multiscale semantic segmentation algorithm was carried out on the UAV pavement imagery for classification. Three principal components obtained, after dimensionality reduction in the multispectral imagery, were combined with deep abstract spatial features from the CNN and fed as inputs to the SVM classifier for pavement surface classification into cracks, potholes, early aging, middle aging and late aging. However, the dimensionality reduction in multispectral imagery demands additional processing. Moreover, low-flying UAVs may not be a practical approach for dense-traffic roadways.

#### 2.2.2. Smartphone-Based Data Acquisition

Ref. [36] carried out an exhaustive review on road surface anomaly detection using smartphone sensors, while highlighting various issues and challenges in the current approaches, and identified research gap areas in the domain. The factors affecting anomaly detection, such as smartphone orientation, mounting location, vehicle suspension and speed dependency, were also investigated. The existing approaches were compared using five primary criteria: sensor data collection, pre-processing, processing, post-processing, and performance evaluations. The major focus of the review was on data collection and processing, rather than result-oriented methodologies, to perform anomaly detection. Ref. [37] performed a smartphone-based pavement analysis to classify the road surface conditions using an accelerometer, gyroscope and GPS data. Multiclass supervised ML techniques were applied using features from all three axes of the smartphone sensors. The classification was focused on three main classes- smooth roads, potholes, and deep transverse cracks. The results confirmed a higher performance than models using single-axis features.

A comparative analysis between the vibration-based method using a smartphone accelerometer and gyroscope and the vision-based method using video processing was performed by [38]. It was observed that vision-based methods outperform the vibration method. However, the latter is sufficient for routine monitoring purposes, while the vision-based method was apt for detailed analysis. Ref. [39] studied and experimented with an ML-based method for the identification and classification of a bottom-up cracked pavement based on vibro-acoustic signatures from roadside sensors. Different ML classifiers, such as MLP, CNN, RFC, and SVM, were used and compared, showing great accuracies for a specific vibro-acoustic signature on concealed cracked road pavements. Ref. [40] studied the vibration-based sensors and GPS embedded in smartphones for automated pothole detection. Data was collected using a customized mobile application installed in dedicated vehicles. A series of image processing techniques were applied, and the extracted features were fed to various ML classifiers to identify the best approach. It was observed that the time and frequency domain features outperformed when identifying potholes, and the RF classifier exhibited the best performance amongst other classifiers, which was evaluated against various road types to check its robustness.

#### 2.2.3. Ground Robot-Based 3D Data Acquisition

In contrast to the traditional 2D and manual methods, Ref. [41] proposed a 3D laser-based pavement scanning and automatic defect detection approach to identify micro (cracks) and macro (deformation) defects. A sparse processing algorithm to extract crack candidate points and deformations support points were designed. The algorithm successfully obtained an accurate location and the classification information of defects with a detection accuracy above 98%. Ref. [42] defined an ML-based system for the acquisition and intelligent classification of potholes and cracks in pavement surfaces. A Kinect device was used to obtain 3D point-cloud structures of pavements and retrieve their physical properties. The system could successfully classify cracks and potholes, along with the computing of the physical properties of pavement depressions, such as the length, width and depth. Ref. [43] created a method for detecting road damage by using a laser scanner mounted on a mobile mapping system to generate 3D pavement images. An attempt was made to segment the pavement using a series of ML techniques. Ref. [44], in their work, developed a computationally efficient pothole-detection algorithm based on disparity maps and disparity transformation algorithms. The detection of potholes was finally achieved by a comparison between actual and modelled disparity maps. The point clouds of potholes were extracted thereafter. The authors created three datasets for stereo vision-based pothole detection, and the algorithm achieved an overall accuracy of 98.7%.

Ref. [45] proposed a fully automated LiDAR-based, two-fold RCM assessment over highways and airport runways using a mobile mapping system. Firstly, a fully automated algorithm was proposed to detect and locate pavement distress from the 3D point cloud, followed by characterization by severity in terms of the depth below the road surface, surface area at different depths and filling volume of the detected potholes. The accuracy of the point clouds was determined to be ±1–2 cm and elucidates the capability of LiDAR-based mobile mapping systems to precisely detect locations with pavement distress of varying severity and different causes. However, the entire setup of the mobile mapping system turns out to be an expensive affair. Ref. [46] developed a pavement evaluation method using mobile mapping systems (MMSs) for automatic detection by spatial frequency analysis of 3D point-cloud data acquired by laser scanners mounted on MMSs. Pavement distresses, such as rutting, flatness and potholes, were detected, along with the crack percentage. The choice of platform and sensors is totally dependent on the specification of a task at hand, taking into consideration the scope, frequency of operation, area in question, extent of detail, budget and strategic importance. A comparative summary of each platform’s usability in the field of pavement distress evaluations is listed in Table 4.

### 2.3. Vision Data-Based RCM Using Deep Learning

**Figure 2:**
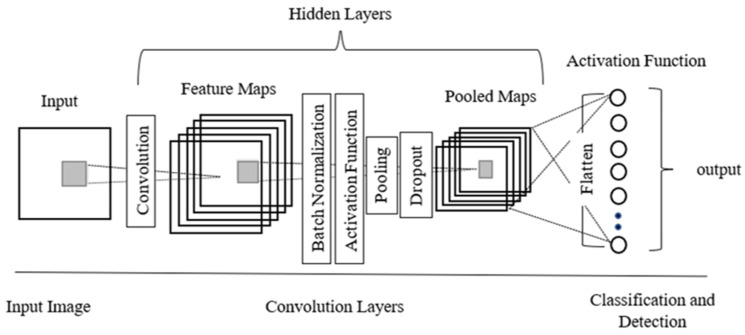
A schematic representation of a CNN.

DL methods have become the most extensively used computational approach in the field of civil engineering and intelligent transportation systems [47,48], attaining outstanding results in RCM. One of the benefits of DL is its ability to learn large quantities of data. With the rapid development of DL techniques, deep convolutional neural networks (DCNNs) have gained paramount significance in carrying out vision-based tasks. In comparison to the conventional handcrafted feature-based techniques, the DL-based techniques learn multi-level image features in detail, which are more descriptive than the handcrafted features. A typical CNN architecture is created using several layers, such as the input layer, hidden layers comprising convolution, pooling and activation functions resulting in feature maps, and the output layer. A deep CNN is characterized by an architecture composed of many layers. Auxiliary layers, such as dropout and batch normalization, are also incorporated within the aforesaid layers as per the necessity of purpose. A schematic representation of a CNN is shown in Figure 2. The first layer of a CNN is the input layer, which is a 2D representation of the input image. The convolution layer performs an element-by-element dot product between a subarray of an input array and a receptive field, followed by a summation of the multiplied values to which the bias is added. This layer is responsible for the reduction in the input data size and subsequent reduction in the computational cost. Batch normalization is performed to re-center and rescale the inputs by normalizing the data distribution. It plays a significant role in smoothening the loss function by optimizing the model. The activation function imparts nonlinearity to the model for semblance to complex real-world problems. The pooling layer is another significant aspect of the CNN responsible for down-sampling as it reduces the spatial size of an input array. A dropout layer can be added to the CNN as an optimization layer to handle complex models that might be prone to overfitting. All these layers, excluding the input layer, comprise the hidden layers of the CNN whose output is fed to fully connected layers resulting in the classification and detection post-application of a suitable activation function.

In general, vision-based DL methods undertake three major pattern recognition tasks: classification, object detection and image segmentation. Classification determines the presence of objects in an image or video. It refers to training DL models to find the classes (pavement distress types) that are present. Classification is useful at the binary level of decision making, whether an image contains the desired object/anomaly or not. A distinct task from the classification is localization, which determines the position of the classified object. Object detection combines classification and localization to determine the objects that are present and specify where they are in the image. It applies classification to distinct objects and uses bounding boxes to show the detection and location of an object. Image segmentation divides an image into regions, extracting potentially meaningful areas for further processing, dependent on its shape and border, such as classification and object detection. The goal of image segmentation is to highlight foreground elements and make it easier to evaluate them. Image segmentation provides pixel-by-pixel details of an object, making it different from classification and object detection. Thus, object classification identifies the category of objects in the image, object detection identifies the category and location of objects with rectangular bounding boxes, and segmentation predicts the categories of each pixel and distinguishes the object instances.

In this paper, the literature review in the following section is catalogued under DL as classification, segmentation and distress detection, and further sub-categorized under data acquisition platforms. It is important to emphasize here that DL-based classification and segmentation methodologies in recent years were restricted to data acquired using ground-based sensors, while UAV and smartphone-based data acquisition platforms find major applicability in areas related to distress detection in RCM, rather than classification and segmentation.

#### 2.3.1. Classifications in RCM Using DL

In contrast to Image Processing Techniques (IPTs), Ref. [49] implemented a vision-based sliding window approach, using deep neural convolutional networks to detect cracks in concrete structures. The high-resolution images were sliced into 40 K images of a 256 × 256 pixel resolution. The method successfully identified the presence of a crack on high-resolution images of the concrete surface and performed well when compared to other IPT algorithms, such as Canny and Sobel edge detection. However, the proposed approach deems to be a computationally expensive approach as it involves redundant scanning through the entire image. A domain adaptation approach by [50] to detect cracks in pavement images provided a simplified vision-based pavement crack detection system. The overall methodology used a truncated VGG-16 DCNN as a deep feature generator, pre-trained on the ImageNet database, to vectorize the labeled pavement images. The cross-domain image classification, a deep transfer learning approach, was an exclusive attempt at automated detection on surfaced pavement images owing to varied surface characteristics. A comparison with existing ML models elucidated the high performance of neural networks in RCM. Ref. [51] presented the German Asphalt Pavement distress dataset for training and evaluation of existing DL models. The authors went a step further by evaluating an image processing toolbox for road pavement surface-crack distresses with shallow and deep neural networks. The authors summarized the results by confirming satisfying detection results by DL approaches, in comparison to conventional computer-vision approaches. Furthermore, the effectiveness of traditional regularization techniques, including dropout, batch normalization, max-norm regularization and weight decay, were assessed. 

An autonomous real-time road crack and pothole detection algorithm was proposed by [52], deployable on a GPU-based conventional processing board with an associated camera. The approach was based on a deep neural network architecture integrated with a pre-processing method to ensure real-time performance. The authors assured that the proposed system could be installed as a plug-and-play module in various autonomous robots and self-driving cars. Ref. [53] proposed the application of CNN on Ground Penetrating Radar images for automatically classifying pavement subgrade defects. Two CNNs called multi-stage CNN and cascade CNN were established to accomplish the tasks that achieved high accuracies. A comparison with ML algorithms further confirmed the CNNs’ robust performance. However, despite the 3D images, the physical characteristics associated with the defects were not calculated. To maximize the evaluation accuracy, an automated pavement evaluation system was proposed by [54]. Two-channel images obtained by overlaying gray-scale (2D) and range (depth) images were fed to a CNN network for image classification. Though the accuracy metrics were not specified, the authors claimed the superiority of the method over conventional ML techniques. Ref. [55] presented an enlarged dataset GAPs v2 as an extension of the GAPs dataset by adding 500 images and providing more refined annotations. Additionally, a CIFAR-like subset of 50 k images was also created for easy comparison and evaluation. Extensive experiments on the extended GAPs dataset were performed using existing DL architectures to understand the role of context in distress detection. Ref. [56] proposed an image-based system for the classification of a speed bump and pothole using a five-layer simple convolutional neural network. The proposed network achieved a classification accuracy of 97.7%. 

Ref. [57] proposed the application of CNNs for pothole detection. A pre-pooling CNN was designed, which added a pre-pooling layer before the first convolution layer to improve the precision of pothole detection. The results demonstrated that the optimized pre-pooling CNN had 98.95% recognition precision on testing data. The stability and comparative study revealed the robustness of the CNN model in real-world situations, such as varying light conditions and pavement materials. Ref. [58] suggested an edge AI-based framework named the vehicular ad hoc network (VANET) for road anomaly detection, such as a pothole, bump, and cracks. Pre-trained DL models, VGG-11 and ResNet-18, were used parallelly for the automated classification and detection of anomalies. The dataset employed was collected from various online sources, which was augmented for better results, and it was suggested that the model’s performance superseded the other techniques used for the detection and classification of road anomalies. Ref. [59] proposed a method for automatic pavement evaluation by the identification, classification and quantification of multiple urban flexible pavement distresses through the application of CNNs. The process involved the concatenation of two CNNs, one that performs identifications with reasonable precision, recall, and F1 score, followed by the quantification of the severity of the predicted distress. Ref. [60] applied a three-layer DCNN to implement crack detection in asphalt pavements by resizing images and classifying them as crack and non-crack. The DCNN was evaluated on two benchmarked datasets and a self-collected dataset, with high accuracy on all datasets.

Ref. [61] proposed a fusion-based pavement damage-detection CNN model for the classification of damages into nine pavement categories using EfficientNet-B4. The fusion was conducted through multi-source sensor information, resulting in a thermal–RGB fused image. It was observed that the model performed better on fused images, in comparison to RGB and thermal images independently. However, in comparison, the prediction performance was found to be better on RGB images for road marking, shadow, and manholes. Ref. [62] proposed an automatic crack classification system by implementing transfer learning on pre-trained networks. ResNet101, GoogleNet and AlexNet were used for crack classification. It was observed that GoogleNet was the fastest to reach maximum accuracy, followed by ResNet101. Ref. [63] proposed a pothole classification system using a fusion of image processing and DL methodology. The process involved the detection of objects other than potholes in pavement images, which were then discarded and the remaining image used for edge detection. Finally, the image post-edge detection was classified using a YOLO classifier to confirm the presence of a pothole.

#### 2.3.2. Segmentation in RCM Using DL

Ref. [64] presented a Feature Pyramid and Hierarchical Boosting Network for pavement crack segmentation and a novel crack measurement technique. The crack detection was formulated as a pixel-wise binary classification task. The method, along with other ML and DL algorithms, was compared and evaluated in relation to five datasets to demonstrate the superiority and generalizability of the method. The authors also presented a pixel-annotated crack dataset of asphalt pavements named Crack500. Ref. [65] presented a road crack segmentation method based on generative adversarial networks (GANs) based on U-Net [66]. The GAN [67] is a special type of neural network architecture used to discriminate between real and fake images, until it is unable to distinguish the real from the fake. The evaluation of the model was performed on three datasets, of which two were public, and one was customized. The model performed moderately on the public dataset with a maximum F1 score of 77.3% and did not obtain good segmentation on a custom dataset. Ref. [68] implemented two DL networks, an improved CNN with structured prediction and an FCN, to detect cracks on pavements with different severity levels. The classification of the cracks was based on crack severity, with an F1 score of less than 70% for both methods. Moreover, the Laser Crack Measurement System software was used, which employed laser intensity to compute the depth of the cracks. The depth factor played a role in determining the severity level during the annotation process. 

Ref. [69] proposed a conditional Wasserstein Generative Adaptive Network (cWGAN) [70] based method, ConnCrack, to the inspect cracks on road surfaces using a cost-effective commercial grade sports camera. This method performed pixel-level crack detection and provided a novel algorithm based on a depth-first search to determine the optimal crack connectivity map. A pixel-level annotated dataset, EdmCrack600, with 600 images was created for public use by the authors. However, in this study, the pixel-level masks were not transformed to the physical properties, such as the width or length of the cracks. Additionally, the proposed method was a data-starving model with training on three datasets (ImageNet, Crack Forest Dataset, and EdmCrack600). Ref. [71] proposed CrackU-net, a crack extraction method from pavement images, regardless of noise levels, background conditions and image quality. The method was based on deep neural networks for pixel-wise crack detection. CrackU-net was trained on images collected by high-speed vehicle-mounted cameras and smartphones and exhibited a high accuracy of 99%.

Ref. [48] proposed a segmentation RCNN model based on Faster RCNN [72] and FCN for RCM. While Faster RCNN performed the feature extraction, classification and localization of distresses, FCN performed a pixel-wise semantic segmentation to provide morphological information on each distress. The result provided a real-time pavement inspection technique, with an average computation time of 34 ms/frame and overall precision and recall of 91.5% and 90.5%, respectively. Ref. [73] proposed a deep generative adversarial network, named CrackGAN, for pavement crack detection. The network promotes crack-patch-only supervised generative adversarial learning for end-to-end training and solves the “All Black” issue existing in FCN-based pixel-level crack detection. The model was trained on small image patches, but can handle all-size images by feeding a bigger-size crack image into an asymmetric U-shape generator. The proposed approach was validated using four crack datasets and achieved good performance in terms of efficiency and accuracy. 

An attempt to automate pavement distress classification based on pixel-level segmentation was performed by [74] using U-Net and Resnet architecture. A dataset was prepared from over a 350 km stretch of asphalt pavements in China along with data augmentation, resulting in over 10,000 images of six types of distresses. Binary classification (distress or non-distress) was performed, resulting in a high accuracy of 97%, while the multi-classification accuracy was fairly low. Ref. [75] proposed the Mask RCNN [76] approach for the pixel-wise segmentation of the Region of Interest in pavements for pothole detection and area calculations. The database was manually collected and annotated with an overall accuracy of 90% for a computed area of a pothole. Ref. [77] proposed a novel DL segmentation approach based on synthetic training data generation for the segmentation of cracks in road pavement images. The synthetic data repository was created using three publicly available datasets: KITTI and Cityscapes for the images of road scenes, and Crack Forest Dataset, as a source of pixel-level labeled cracks. Segmentation was implemented using Mask R-CNN and U-Net, showing good results.

A grid-based crack detection model was proposed by [78] using a segmentation CNN approach. Each image was divided into three grids of different sizes, namely 10 × 10, 20 × 20 and 30 × 30, as inputs to the segmentation model to classify crack or no crack. Grids with cracks were segmented into binary images using the entropy thresholds, which were then used to calculate the crack widths. Recently, Ref. [79] performed an extensive study by incorporating stereo vision and DL to efficiently perform crack and pothole segmentation using an automated pixel-level detection framework for RCM. The multi-view stereo imaging system was used to establish the datasets containing color images, depth images and color-depth overlapped images. A modified U-Net was implemented and tested on asphalt roads with millimeter-level accuracy. The 3D crack segmentation model outperformed the other models in terms of the inference speed and segmentation accuracy. Additionally, a highly accurate automated pothole volume measurement method based on a segmentation map was proposed to evaluate pothole severity. 

#### 2.3.3. Damage Detection in RCM Using DL

Ref. [80] proposed the use of the Faster R-CNN algorithm on a customized dataset for damaged pavement detection, which was divided into six distinct categories: lateral crack, longitudinal crack, pothole and separation, alligator crack, well cover without damage and damage around the well cover. The proposed CNN architecture showed high accuracy and stability in locating and identifying the cracks in the dataset. Banking on the advantages of automatic feature extraction by CNNs, Ref. [81] presented a framework based on DL to automate the roadway condition assessment. A CNN was designed to classify four types of pavement cracks, namely, longitudinal, transverse, alligator and potholes, and the crack detection dataset was created from images taken from search engines. The overall accuracy of the proposed CNN was 76%, which was justified as reasonable when compared to the methodologies limited to binary classifications. 

Ref. [82] analyzed the feasibility and accuracy of thermal imaging in the field of pothole detection. The work involved the acquisition of thermal and visual images of potholes under various conditions and the application of augmentation techniques, followed by the implementation of convolutional neural networks on the images. A comparison between the customized and pre-trained models revealed the best accuracy of 97.08% with ResNet101. Ref. [83] implemented a novel method for pothole detection by integrating images with mobile mapping point-cloud data. This method first detected the location and 2D edge of a candidate pothole in the image. The point clouds were then used to differentiate between potholes and patches by calculating the mean depth of the candidate pothole. The geometric accuracy of the pothole extraction was evaluated using a simulation experiment, showing that the mean size accuracy was ~1.5–2.7 cm.

Ref. [84] utilized existing DL methods to carry out a hotspot analysis on urban road networks, highlighting pavement distress types and associated severities in terms of remedial actions that were either necessary or not. Efforts were also made to outline approaches to using the analysis for the continuous monitoring of pavement health. Damage detection models were accurately able to highlight the location and assess distress severity. However, the severity analysis was limited by human bias carried out during data annotation, rather than post-detection employing mathematical calculations. To assess the workability of DL models on Google Street View images, Ref. [85] implemented two classical DL frameworks for pavement condition assessment on a customized labeled dataset. The images were manually annotated, and the distresses were classified into nine categories, namely, reflective, transverse, block, longitudinal, alligator, sealed transverse, sealed longitudinal, and lane longitudinal cracking, along with potholes.

Ref. [86] developed stereo-vision techniques for the automatic identification of potholes. Transfer learning-based DL methods for segmentation (Mask R-CNN) and localization (YOLOv2) were also used for pothole detection, suggesting good precision results and real-time applicability, respectively. Ref. [87] proposed a 2D vision-based pothole detection method using two-stage CNNs that focused on the discriminative regions in the road instead of the global context. The approach was based on using two CNN subnetworks, a localization network to find the regions likely to contain potholes based on heat maps and a second CNN for classification, which feeds on the candidate regions from the localization network. The experiments on the pothole dataset showed that the proposed method achieved high precision and recall, and outperformed most existing methods. However, this approach turned out to be expensive as the localization network added prior to the classification increased the computational time. 

To classify the road surface characteristics and destabilizations produced by pavement distresses, and man-made obstructions, Ref. [88] proposed a DL approach to identify the kinds of road surfaces and distinguish the stability events produced by potholes from the stability events produced by other man-made structures or driver actions. A mobile application was used to record the vibration and GPS information. DL models, such as CNN, Long Short-Term Memory (LSTM), and Reservoir Computing were trained to identify the types of road surfaces and different stability events. The models performed well with the best accuracy of 85% for road surface classification and 93% for stability event classification by the CNN. Ref. [89] extensively evaluated the performance of eight DL models on the RDD2020 dataset [90], and presented a modified dataset based on the same. The study recognized the single-shot detector (SSD-Inception V2), and faster R-CNN-Inception V2, as the best road-damage-detection approach in terms of accuracy and image processing time.

Ref. [91] proposed a solution to detect dry and wet potholes using smartphone sensors and camera images. Twelve different sensor values were collected and manually annotated to perform pothole detection using ML algorithms. The captured images were examined for potholes by applying Mask R-CNN and U-Net segmentation algorithms. Both sensor and camera-based methodologies provided promising results. Ref. [92] proposed a RCM system that can be integrated with road-damage acquisition systems and presented an asphalt pavement dataset with over 45,000 instances of various distress types. RetinaNet architecture with VGG16 as the backbone was used, promising a better performance than other object detection models. The light memory footprint allows the model to have an easy integration into mobile systems.

Ref. [93] presented an RCM method based on Faster R-CNN to recognize and locate cracks, potholes, oil bleeding and dot surface autonomously. A total of 20 Faster R-CNNs were trained and tested on 6498 pavement images, with the performance of the optimal one having accuracy rates, recall rates and location errors of 90.4%, 89.1% and 6.521 pixels. In comparison to the CNN and *K*-value method, the optimal Faster R-CNN located pavement distresses with more precision [94] proposed a methodology for automatic pavement image distress detection and classification using CNN models and a low-cost vehicle-mounted high-definition camera. The pavement distress types were categorized as linear or longitudinal crack, network crack, fatigue crack or pothole, patch, and pavement marking. The detection rate and classification accuracy of the proposed approach with the trained CNN model reached 83.8% over the test set. A sensitivity analysis was also carried out for evaluating the different regularization techniques and data generation strategies. 

Ref. [95] developed a DL model, RoadID, to detect multiple pavement distresses. The distress dataset contained over 44 k images, 14 k damages, and 8 distress types—crack, net, pothole, patch crack, patch net, patch pothole, hinged joint, and manhole. The model successfully located and identified the pavement damages with a mAP of 85.94%. Ref. [96] proposed a pothole detection method using a modified VGG16 (MVGG16) network as a backbone for the Faster R-CNN. The key feature of the MVGG16 network was its ability to capture more contextual information by increasing the dilation rate of the convolution. A comparison of the performance of YOLOv5 and Faster R-CNN with different backbones proved that the MVGG16 network as the backbone of the Faster R-CNN provides a better mean precision and shorter inference time than using other backbones. Ref. [97] performed a classification of the pavement cracks into longitudinal, transverse, alligator cracks and potholes using the DL model YOLO v3 for the localization and detection of cracks with a precision of 0.7 and an average IoU of 50.39%.

In view of inadequate large-scale data in civil engineering projects, Ref. [90] presented RDD2020 by extending the existing RDD2018 dataset using GAN-based data augmentation. PG-GAN and Poisson blending were used to generate realistic road damage images, which fared well in the visual Turing test. The pothole dataset was increased to 2000 images, and DL methodologies performed reasonably well on the augmented dataset. Ref. [98] proposed a DL methodology to automatically localize diseased areas in pavements using a novel approach named the Iteratively Optimized Patch Label Inference Network. The method involved training image patches obtained by slicing high-resolution images and inferring the patch labels using an algorithm based on the label of the high-resolution image. The authors also released a novel, large-scale RCM dataset, CQU-BPDD, involving various pavement diseases. 

Ref. [99] implemented a highly accurate pavement defect and cleanness inspection system using a DL-based framework in the pavement-sweeping robot Panthera. A lightweight DCNN model was developed and trained on 6000 pavement defect and garbage images, which took approximately 132.2 milliseconds for detecting both pavement defects and garbage. Moreover, the geotagging of the pavement defects helped in mapping the defects. In their work, Ref. [100] suggested that pothole detection categorized on specific road types produced better results. The proposed model first classified the road surface as asphalt, unpaved and paved using a CNN algorithm, followed by three YOLO v3 models, respectively, for different road surfaces to detect potholes. Public datasets were used for training, and augmentation was performed to improve the model accuracy. The authors went a step further by geotagging the images during detection and updating a database to mark the potholes on maps’ pothole tracking. The geotagged images were used to identify the street and keep an account of distress conditions in that area. 

Ref. [101] presented a CNN-based pothole detection method on a small dataset and evaluated the performance of Faster RCNN with YOLOv3. As per the results, Faster RCNN performed better than its counterpart. The accuracy of Faster RCNN was further improved by integrating it with feature extraction using VGG16. The dataset was also increased using augmentation techniques to achieve a better performance. Ref. [102] compared and evaluated various DL object-detection techniques for damaged pavements to detect potholes. The Kaggle and RDD2020 datasets were used for comparison, and it was concluded that YOLOv4 performed the best with a mAP of 0.535. Ref. [103] proposed a CNN-based approach for the automated detection of pavement distresses using images collected from smartphones. The distresses were classified into six categories [90]. The system demonstrated a practical efficiency and detection rate with more than 80% accuracy. Ref. [104] implemented the YOLOv3 DL algorithm for pothole detection with a mAP of 65.65% on 416 × 416 pixel images. The detected potholes were logged, and their locations translated for visualization on Google Maps using Google API.

Ref. [105] studied three YOLO object-detection frameworks to evaluate the best performance for real-time pothole detection. The experiment was conducted on a dataset of 665 images of 720 × 720 resolution using YOLOv4, YOLOv4-tiny, and YOLv5. It was confirmed that YOLOv4-tiny was the best fit for pothole detection with a mAP of 78.7%. Ref. [106] implemented a single-stage object detector called RetinaNet to detect and classify fatigue, longitudinal, and transverse cracks having different severity levels using an automated road-scanning vehicle with two cameras. The authors declared that the accuracy of detection and classification was highly dependent on the amount and quality of training images, and the detection error exponentially decreased with an increase in the number of training images. The trained network model achieved a detection accuracy of 84.9% on images annotated with a crack type and severity level, while achieving an accuracy of 89.1% when only considering the crack type. Ref. [107] proposed a novel Faster R-CNN approach to localize and classify the cracks and damages in pavements. The Faster R-CNN approach used pre-trained models, such as VGG16 and ResNet152. A dataset of 3533 images was manually collected and categorized into alligator crack, linear crack, nonlinear crack, damage and non-crack image. The overall accuracy of the model with VGG16 was observed to be 87%, while with ResNet152 was 90%.

##### UAV-Based Data Acquisition

Ref. [108] designed a distributed platform for pavement damage detection using drones and a multi-agent architecture PANGEA (Platform for Automatic coNstruction of orGanizations of intElligent Agents). The YOLOv4 classifier was customized to achieve promising results with an accuracy of 95%. The images acquired by drones used in the dataset have been published for use by the scientific community. Ref. [109] implemented a real-time drone-based DL model to detect the cracks and potholes in pavement images. The method involved identifying the yellow lane of the road for drone-flight autonomous navigation, while concurrently performing real-time road crack and pothole detection using the robot operating system within the UAV. The CNN achieved an F1 score of 85.78 and 94.04 for cracks and potholes, respectively. Ref. [110] studied drone-flight settings for optimal pavement image quality and provided an open dataset unmanned aerial pavement dataset (UAPD) for distress analysis with six distress types. Three object-detection algorithms were used to train and test the UAPD. The prediction performances of the three algorithms were compared to YOLOv3 as the best, having a mAP of 56.62% and the ability to recognize cracks in different environments, including shadows, trees, and pavement markings.

##### Smartphone-Based Data Acquisition

Ref. [111] prepared a large-scale road damage dataset with eight damage categories using a smartphone installed on a car. The training and evaluation of a lightweight CNN-based damage detection model on the proposed data set were undertaken, with an assessment of accuracy and runtime speed on a GPU server as well as a smartphone. Finally, the road damages were classified into eight types by applying the proposed object detection method. The road damage dataset is a breakthrough dataset that standardizes and provides a platform to researchers for the comparison and evaluation of state-of-the-art DL models. However, the model used for road damage detection does not provide a high accuracy for different classes of road damage. Ref. [112] proposed an IoT-based end-to-end system named PotSpot for monitoring and mapping potholes. An Android application was built to perform CNN-based pothole detection integrated with Google Maps API for a real-time pothole-marked map. The performance of the model was evaluated on a real-world road image dataset with an accuracy of 97.5%. Ref. [113] implemented a self-driving car model that can avoid potholes using a vision-based CNN approach. The end-to-end approach does not detect potholes as bounding boxes, but rather avoids potholes by predicting the vehicle parameters for driving decisions and the automatic control of the car. The game simulator was used to collect driving data and perform training and testing.

Thus, vision-based classification, segmentation and detection have largely contributed to RCM, allowing distress detection, monitoring and analysis. Each of these DL methodologies has its own strengths and limitations, as summarized in Table 5. It is important to note that data is an important consideration in determining the performance of DL models; thus, various data augmentation techniques have been implemented to address class imbalance issues and increase the dataset size for better training and evaluation. To facilitate the research community in future endeavors, a list of openly available data sources has been compiled, as illustrated in Table 6.

## 3. Future Research Directions

Endeavors have been made to bridge the gaps and successfully implement DL algorithms in RCM using data acquired from sensors. In addition to the breakthrough results attained by researchers in this field, there exist several challenges that are listed below:

- Quality and quantity are often considered as the keys to a balanced dataset and contribute to high performance in DL models. However, in spite of data augmentation techniques resulting in large pavement datasets [91], the performance of different DL models is not satisfactory. Thus arises a need to quantify the size of multi-class samples in a dataset and identify appropriate augmentation algorithms and methods to ensure a balanced, all-encompassing dataset.
- Standardization of distress types provides a comprehensive evaluation platform to identify the best approach. However, it is observed that there exists no standard nomenclature for pavement distress annotations in the publicly available datasets, resulting in an ineffectual pavement distress analogy.
- DL models demand large storage space. The size of high-performing models and their computational requirements pose a challenge to their usability in real-time scenarios.

To streamline the process above and beyond the existing challenges, the following directive actions need to be brought into effect.

(i) Real-time processing: on-board processing on UAVs and other platforms demand a lightweight though efficient detection model for the real-time assessment of pavements. There is a need to develop computationally reasonable and cost-effective models that can be deployed on sensor platforms to provide prompt results and avoid processing delays.

(ii) Data standardization: with large amounts of work being conducted in the development of a pavement management system, there is a need to standardize the data requirements to benefit cumulatively from the contributions made by the research community in the field. Data regulations can ensure a global collaboration towards a common goal, resulting in a robust solution to the problem.

(iii) Characterization of distresses: the autonomous detection of distresses has paved the way beyond the limitations faced by conventional systems. However, a step further in this direction that quantifies the extent of damage can help agencies to prioritize the regions demanding immediate attention. Thus, the physical characterization of distresses can streamline the evaluation system by working towards a cohesive solution.

(iv) Crowdsourcing platform: with data no longer being a limitation and ease of access to technology, a crowdsourcing platform can be developed for reporting distresses. This will add to the collective database and a robust system that can recognize and report the different distress types.

(v) Multidisciplinary research: civil engineers, computer scientists and data analysts can come together by contributing their expert domain knowledge to build an exhaustive distress evaluation system. A proper understanding of the pavement structures and their distresses can contribute to high-quality data resources and help in designing high-performance DL models.

(vi) High computing resources: DL models are data hungry and need high-performance computing resources. Cloud Computing and Edge Computing resources, including Internet-of-Things sensors, 5G and 6G, can be instantly provisioned with the ability to scale up and down. This will also allow systematic computing on a long-term basis devoid of memory and compute resource limitations.

(vii) Integrated system: innovation in system integration is another area on which research needs to be focused. The end-users are mostly government agencies and can benefit more from a package that delivers an end-to-end solution.

## 4. Conclusions

In this paper, the existing research work in RCM using next-generation sensors and AI methodologies was extensively reviewed and compared. The existing approaches are evaluated based on the data acquisition platforms under various AI approaches, especially DL for classification, segmentation and object detection. Data acquisition systems, which are a combination of non-intrusive sensors and their platforms, are at the center of the RCM system and involve the collection of 1D data, 2D visual data or 3D depth data. Every platform has its own advantages and limitations and complements each other’s usability. For the tasks involving a simple classification of distresses, RGB sensors are a good option, while for a detailed study involving the various characteristics of distresses, LiDAR, laser, thermal or GPR sensors can be used. It is also necessary to appreciate the value and importance of data, along with the need to understand with clarity the definition of data. Whether the problem deals with classification, detection, or localization in RCM, at the core of every DL vision algorithm is a large collection of labeled images. Thus, another important step is the data annotation of collected data as ground truths, a crucial step in determining the accuracy of any DL model.

Developing DL models that accurately identify and characterize pavement distress is a challenging task. Overall, the CNN-based DL classification models show very high accuracy in detecting the presence or absence of multiple pavement distresses, and are better than conventional computer vision approaches in terms of performance; however, they fail to specify the location of distresses and thus lose their applicability in real-time scenarios. Over the course of time, CNN algorithms involving intelligent techniques to extract pavement distress features, such as transfer learning, pre-pooling layers, parallel CNNs, multi-stage CNNs, multi-source sensors, and IPTs integrated with CNNs, have been adopted to improve the existing performance of the DL classifiers.

Segmentation, which performs pixel-level classification employing CNNs, is another approach used in pavement distress analysis and can be used to extract the morphological characteristics of the distresses. However, not much work has been conducted in retrieving the physical characteristics of pavement damages, attributed to noisy post segmentation. Different approaches, such as connectivity maps and GANs, are being used to enhance segmentation outputs. Extensive work is being conducted in this area to precisely measure distress dimensions in terms of pixels; however, measurement accuracy, a decisive factor in determining the distress severity, is a contentious issue faced by many researchers.

Distress detection that involves classification and localization shows good results with 2D data and promises detailed analysis with 3D data, but not much work related to the measurements of distresses has been undertaken in this area. The detection of pavement damages helps in localizing the defects and has led many researchers to overlay this information on maps for navigation purposes. Innovative methods, such as thermal imaging, point cloud data, stereo-vision analysis, location-aware CNNs, mobile applications and UAV-based models for real-time assessment, to name a few, have been applied by many researchers to enhance the performance of existing CNN models. It has also been observed that CNN models have achieved a high performance in localizing distresses as a single class, when compared to localizing distresses under various classes. The performance of DL algorithms with advanced contributions from the scientific world and standardizations will continue to validate the use of DL approaches in the field of RCM. CNNs are currently the most reliable method to monitor the pavements for distresses, and the further integration with innovative technologies, affordable sensors and platforms shall encompass the futuristic, fully automated RCM systems.

## Figures and Tables

**Table 1:** Types of common pavement distress (Miller and Bellinger 2014).

Distress Type		Severity Levels	Causes
Crack			
Alligator	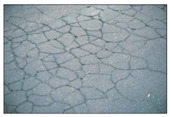	Low (no or few interconnections) Moderate (forming a pattern) High (severely spalled)	Repeated traffic loadings
Block	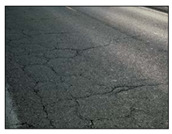	Low: width ≤ 6 mm Moderate: 6 mm < width ≤ 19 mm High: width > 19 mm	Repeated traffic loadings
Longitudinal	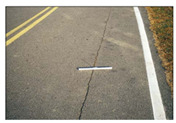	Low: width ≤ 6 mm Moderate: 6 mm < width ≤ 19 mm High: width > 19 mm	Poor joint construction or location
Transverse	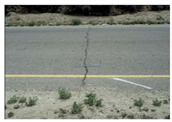	Low: width ≤ 6 mm Moderate: 6 mm < width ≤ 19 mm High: width > 19 mm	Axial loading or temperature change
Pothole and Patch			
Pothole	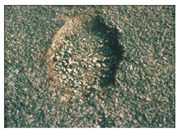	Minimum diameter of 150 mm Low: depth ≤ 25 mm Moderate: 25 mm < depth ≤ 50 mm High: depth > 50 mm	Water infiltration into cracks
Patch	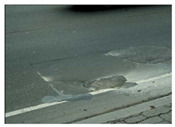	Surface ≥ to 0.1 m^2^ Low: rutting ≤ 6 mm Moderate: 6 mm < rutting ≤ 19 mm High: rutting > 19 mm	Lack of preventative maintenance
Surface Deformations			
Rutting	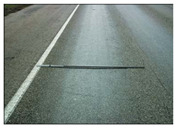	Not applicable	Heavy load, wheel path, poor mix
Shoving	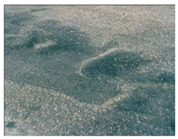	Not applicable	Plastic movement of pavement surface, weak subgrade, improper rolling
Surface Defects			
Bleeding	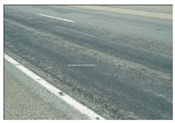	Not applicable	Excessive binder, low air-void content
Ravelling	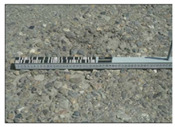	Not applicable	Weather, installation, aggregate separation, mechanical dislodging
Polished Aggregate	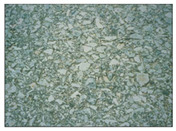	Not applicable	Aggregate with insufficient flakiness and excessive friction on the road by vehicles

**Table 2:** A comparative evaluation of the smart sensors used in RCM.

Key Variables	Camera	Laser	GPR	Thermal	Vibration
Technology	2D imaging	3D construction of image using reflection	Radio waves to explore underground surface; creates 3D image of sub-surface	Based on the change in temperature of surrounding objects using infrared waves	Accelerometers, gyroscope, and GPS readings
Processing	Complex image-processing algorithms	Collection of 3D point cloud	Collection of depth images and simulation data required	Collection of heat variation of surface	Readings are directly used
Real-Time Application	Processor dependent	Yes	Yes	Yes	Cannot be used in real-time detection
Sensing Time	While approaching distress	While approaching distress	While approaching distress	While approaching distress	Only after experiencing distress
Characterization of Distress	Based on shape and size	Based on 3D image	Based on 3D image	Based on heat maps	Detection only along wheel path as 1D parameters
Light Sensitivity	Sensitive to illuminance levels, light source position	Not sensitive to light effect	Not sensitive to light effect	Not sensitive to light effect, but surface temperatures	None
Accuracy	Algorithm dependent	High	High	High	Highly susceptible to errors
Resolution	Varying low to high	High-resolution images	Depends on frequency	Needs improvement	-
Processing Time	Data collection and analysis is fast; response time is processor dependent	Data collection is fast and can be collected at speeds as high as 100 km/h	Delayed due to large data processing; however, data collection is automated	Data collection and analysis is fast	Poor as data processing is required
Cost	Economical	High	Highly expensive	Very expensive	Low
Data Type	2D, 3D	3D	3D	2D, 3D	1D

**Table 3:** List of sensor platforms available for data acquisition.

Sensor Platform	Advantages	Limitations
Unmanned Aerial Vehicle 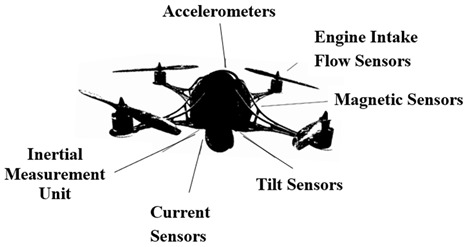	Large FOV. High Resolution. In-depth, detailed data. Ease of deployment and accessibility in hazardous areas. Flexibility for quick inspections.	Payload and memory restrictions. Legislative restrictions.
Ground Vehicle 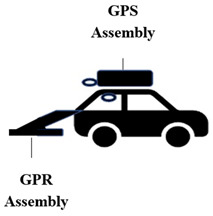	Long span availability. Array of sensors. High-resolution imagery. Highly dense and occluded terrain.	Small FOV.Less cost-effective. High dependency on manpower.
Smartphone 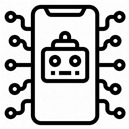	Lightweight technology. Economically viable.	Low-resolution imagery. Limited by RGB data.

**Table 4:** A comparative summary of the data acquisition platforms used for RCM.

Platforms	Advantages	Limitations
UAVs	Well-matched for aerial reconnaissance. Unhindered large field of view. Allow for navigation through difficult terrains. Facilitate safe and quick inspections.	Unsuitable for RCM in dense traffic roadways. Limited by weather conditions, such as wind speed and harsh climate.
Smartphones	Lightweight sensors. Ease of employability due to their size. Stand-alone (hand-held device) data acquisition method and can be easily installed on vehicles.	Limited by image resolution. Suffer noisy data due to external factors.
Ground robots	Extensive usability in dense traffic areas. Scalable platforms for multi-array sensors. Find widespread serviceability in RCM.	Limited by a small field of view. Poor cost-effectiveness in terms of long hours of operation and resources.

**Table 5:** The advantages and limitations of DL methodologies in RCM.

DL Methodologies	Advantages	Limitations	Accuracy
Classification	Better than conventional ML approaches in terms of performance.	Demands training on large volumes of data. Very high-resolution images subjected to stitched patches with distresses. Thus, the results are discontinuous and have ambiguous structural semantics.	Ranges from 90–97%
Segmentation	Performs pixel-level classifications. Pixel-wise class assignment allows an in-depth analysis of an image. Helps in determining the morphology of the distress.	Demands training on large volumes of data. Requires post-processing algorithms to extract exact and smooth shapes from pixelated outlines. Results are prone to noises. Most of the studies seldom focus on studying the physical characteristics associated with the defects, such as width and length.	Ranges from 70–99%. Higher accuracies observed in single-class segmentation problems.
Detection	High accuracies in pavement distress detection. Provide classification as well as localization of defects. Allow the mapping of defects. With technologies, such as depth measurement systems using LiDAR, and laser, and point clouds, the measurement of physical characteristics of pavement distress is possible.	Demands training on large volumes of data. Physical characteristics of pavement distresses remain a gap, when limited to 2D data evaluation.	Ranges from 70–97%. Higher accuracies observed in single-class object detection, when compared to multi-classification and detection.

**Table 6:** List of the open-source databases on pavement images.

Reference	Name of Database	Type	Number and Type of Images
[51]	GAPs	Asphalt	1968 grayscale
[90]	RDD2019	Asphalt	26,336 RGB
[41]	Crack500	Asphalt	500 RGB
[41]	GAPs384	Asphalt	384 grayscale
[114]	CrackTree200	Asphalt	200 grayscale
[115]	Crack Forest Dataset	Asphalt	118 grayscale
[116]	AEL	Asphalt	58 grayscale
[117]	Deep Crack	Concrete, asphalt	537 RGB
[55]	GAPs v2	Asphalt	2468 grayscale
[116]	AigleRN	Asphalt	38 grayscale
[85]	Pavement Image Dataset	-	7237 RGB
[108]	-	Asphalt	1362 RGB
[44]	-	Asphalt	630 RGB
[95]	RoadID	Asphalt	44,532 RGB
[110]	UAPD	Asphalt	3151 RGB
[98]	CQU-BPDD	Asphalt	60,059 RGB
[69]	EdmCrack600	-	600 RGB
[92]	Road Surface Damage	Asphalt	18,345 RGB

## Data Availability

Table 6 provides the link to all the datasets that are publicly available.

## Abbreviations

AI	Artificial Intelligence
DCNN	Deep Convolutional Neural Network
DL	Deep Learning
FCN	Fully Convolutional Network
FOV	Field of View
GAN	Generative Adversarial Network
GPR	Ground Penetrating Radar
GPS	Global Positioning System
IoU	Intersection over Union
IPT	Image Processing Technique
IRI	International Roughness Index
LiDAR	Light Detection and Ranging
mAP	Mean Average Precision
ML	Machine Learning
RCM	Road Condition Monitoring
RDD	Road Damage Dataset
R-CNN	Region Convolutional Neural Network
RF	Random Forest
SVM	Support Vector Machine
UAV	Unmanned Aerial vehicle
YOLO	You Only Look Once
